# 

**Published:** 1921-11

**Authors:** 


### ANNOUNCEMENTS

### Ohio State Dental Society

The fifty-sixth annual meeting will be held in Columbus, in the Memorial Hall, December 6-8, 1921. A fine programme is being prepared and a cordial invitation is given to all members of other state societies to attend all sessions.—F. R. Chapman, *Secretary.*

### Minnesota State Dental Association

The Minnesota State Dental Association will hold its thirty-ninth annual meeting in Minneapolis, February 22-25, 1922. The meetings will be held in the Armory Building.

### The Dental Protective Association

The annual meeting will be held in Chicago, at the Palmer House, the 19th of December, at 4 o'clock, P. M. Report of officers; a Board of Directors will be elected, and other business will be transacted. All members who can are urged to attend.—J. G. Reid, *President;* D. M. Gallie, *Treasurer;* E. W. Elliot, *Secretary.*

### United States Civil Service Examination

### *Dental Assistant*

*Applications will be rated as received until January* 31, 1922—The United States Civil Service Commission announces an open competitive examination for dental assistant. Vacancies in the Public Health Service throughout the United States at $80 a month, without quarters, subsistence, or laundry, and vacancies in positions requiring similar qualifications, at this or higher or lower salaries, will be filled from this examination, unless it is found in the interest of the service to fill any vacancy by reinstatement, transfer, or promotion.

*Bonus*—Appointees whose services are satisfactory may be allowed the increase granted by Congress of $20 a month.

*Citizenship and sex*—All citizens of the United States who meet the requirements, both men and women, may enter this examination; appointing officers, however, have the legal right to specify the sex desired in requesting certification of eligibles.

*On account of the needs of the service, papers will be rated as received and certification made as the needs of the service require. In the absence of further notice, applications for this examination will be received by the Commission at Washington, D. C., until the hour of closing business on January* 31, 1922. *If sufficient eligibles are obtained, the receipt of applications may be closed before that date, of which due notice will be given.*

*Certification*—In filling vacancies in this position, certification will be made of the highest eligibles residing nearest the vicinity of the place at which the appointee is to be employed, except that upon request of the department certification will be made of the highest eligibles on the register for the entire country who have expressed willingness to accept appointment where the vacancy exists.

*Subjects and weights*—Competitors will not be required to report for examination at any place, but will be rated on the following subjects, which will have the relative weights indicated:

| *Subjects* | *Weights* |
|---|---|
| 1. Physical ability | 25 |
| 2. Education and experience | 75 |
| Total | 100 |

*Basis of ratings*—Under the second subject the ratings will be based upon competitors' sworn statements in their applications and upon corroborative evidence.

*Duties*—The duties of this position are to care for dental instruments, to keep equipment and cabinets in order, and to assist the dentist in the preparation of materials.

*Requirements*—Applicants must have completed at least the sixth grade of common school. In addition, they must have had at least one year's private training in a dental office or one year's experience as a graduate or practical nurse assisting a surgeon in the operating room.

*Age*—Applicants must have reached their eighteenth but not their thirtieth birthday on the date of making oath to the application. These age limits do not apply to persons entitled to preference because of military or naval service.

*Retirement*—Classified employees who have reached the retirement age and have served fifteen years are entitled to retirement with an annuity. The retirement age for railway mail clerks is 62 years, for mechanics and post-office clerks and carriers, 65 years and for others 70 years. A deduction of 2½ per cent. is made from the monthly salary to provide for this annuity, which will be returned to persons leaving the service before retirement with 4 per cent. interest compounded annually.

*Photographs*—Applicants must submit with their applications their unmounted photographs, taken within two years, with their names written thereon. Proofs or group photographs will not be accepted. Photographs will not be returned to applicants.

*Medical certificate*—The medical certificate in the application form should be executed by a medical officer of the Public Health Service where practicable.

*Applications*—Applicants should at once apply for Form 1312, stating the title of the examination desired, to the Civil Service Commission, Washington, D. C.; the Secretary of the United States Civil Service Board, Customhouse, Boston, Mass., New York, N. Y., New Orleans, La., Honolulu, Hawaii; Post Office, San Francisco Calif., Denver, Colo.; Old Customhouse, St. Louis, Mo.; Administration Building, Baloba Heights, Canal Zone; or to the

Chairman of the Porto Rican Civil Service Commission, San Juan, P. R.

Applications should be properly executed, *including the medical certificate*, but excluding the county officer's certificate, and filed with the Civil Service Commission, Washington, D. C., without delay.

*The exact title of the examination, as given at the head of this announcement, should be stated in the application form.*

*Preference*—Applicants entitled to preference should *attach to their applications* their original discharge, or a photostat or certified copy thereof, or their official record of service, which will be returned after inspection.

*Issued October 28, 1921.*

### THE FUTURE OF DENTISTRY

### MANY PROBLEMS STILL TO BE SOLVED—RETTER TRAINED PRACTITIONERS NEEDED

Dentistry's progress in the near future will be on the scientific side, according to Dean Eugene H. Smith, of the Harvard Dental School.

"The next decade will undoubtedly see the same sort of advance in dentistry that is taking place today in medicine," says Dean Smith. "Scientific research and scientific knowledge will teach us more about the causes and effects of ailments of the teeth, and about how to prevent them.

"The dental profession reached its peak of sheer technical competence a few years ago, so far as we can see now. The leaders of the profession now show amazing skill in doing the excessively difficult and careful work of repairing and straightening and filling teeth, and while new instruments and apparatus will probably be invented which will enable such work to be done still more effectively, nevertheless it is not along this line that we look for dentistry to make its big advance in the immediate future. The big advance will undoubtedly be on the scientific side.

"There is room for advance in the study of the chemical relation between diet and healthy teeth, and of the relation between decay of the teeth and rheumatism, neuralgia, heart disease of various kinds and many other ailments. At present we know that there is a connection between decay of the teeth and rheumatism, but there is a lot of loose thinking done on the subject, and a lot of teeth are taken out by some dentists to stop rheumatism which oughtn't to be taken out at all. This whole side of dentistry needs to be put on a firmer scientific foundation.

"That is why we now require a year of pre-dental college work before we will admit men to the Harvard Dental School. That is why the dental departments of many universities have joined us in this step. And that is why we expect, in the course of a few years more, to require not one year of college, but two.

"Such an increase in the admission requirements is naturally going to cut down the size of our school somewhat for the present. We have a small first-year class this year, only a fraction of the size of last year's. But we intend to go right on. For only by requiring of the dentist a broader educational background are we going to make it possible to give him in our Dental School the sort of equipment of general medical knowledge which he must have if he is to take part in the new scientific advance of dentistry."

